# Regulation of the Telomerase Reverse Transcriptase Subunit through Epigenetic Mechanisms

**DOI:** 10.3389/fgene.2016.00083

**Published:** 2016-05-09

**Authors:** Kayla A. Lewis, Trygve O. Tollefsbol

**Affiliations:** ^1^Department of Biology, University of Alabama at Birmingham, BirminghamAL, USA; ^2^Comprehensive Center for Healthy Aging, University of Alabama at Birmingham, BirminghamAL, USA; ^3^Comprehensive Cancer Center, University of Alabama at Birmingham, BirminghamAL, USA; ^4^Nutrition Obesity Research Center, University of Alabama at Birmingham, BirminghamAL, USA; ^5^Comprehensive Diabetes Center, University of Alabama at Birmingham, BirminghamAL, USA

**Keywords:** human telomerase reverse transcriptase (hTERT), epigenetics, DNA methylation, histone acetylation, histone methylation, non-coding RNA

## Abstract

Chromosome-shortening is characteristic of normal cells, and is known as the end replication problem. Telomerase is the enzyme responsible for extending the ends of the chromosomes in *de novo* synthesis, and occurs in germ cells as well as most malignant cancers. There are three subunits of telomerase: human telomerase RNA (hTERC), human telomerase associated protein (hTEP1), or dyskerin, and human telomerase reverse transcriptase (hTERT). hTERC and hTEP1 are constitutively expressed, so the enzymatic activity of telomerase is dependent on the transcription of *hTERT*. DNA methylation, histone methylation, and histone acetylation are basic epigenetic regulations involved in the expression of *hTERT*. Non-coding RNA can also serve as a form of epigenetic control of *hTERT*. This epigenetic-based regulation of *hTERT* is important in providing a mechanism for reversibility of *hTERT* control in various biological states. These include embryonic down-regulation of *hTERT* contributing to aging and the upregulation of *hTERT* playing a critical role in over 90% of cancers. Normal human somatic cells have a non-methylated/hypomethylated CpG island within the *hTERT* promoter region, while telomerase-positive cells paradoxically have at least a partially methylated promoter region that is opposite to the normal roles of DNA methylation. Histone acetylation of H3K9 within the promoter region is associated with an open chromatin state such that transcription machinery has the space to form. Histone methylation of *hTERT* has varied control of the gene, however. Mono- and dimethylation of H3K9 within the promoter region indicate silent euchromatin, while a trimethylated H3K9 enhances gene transcription. Non-coding RNAs can target epigenetic-modifying enzymes, as well as transcription factors involved in the control of *hTERT*. An epigenetics diet that can affect the epigenome of cancer cells is a recent fascination that has received much attention. By combining portions of this diet with epigenome-altering treatments, it is possible to selectively regulate the epigenetic control of *hTERT* and its expression.

## Introduction

Telomeres are DNA sequences that cap the ends of chromosomes in order to compensate for the end-replication problem. This end-replication problem is the result of DNA polymerase being unable to reach the end of the lagging strand of chromosomes during DNA replication. Because DNA polymerase cannot reach the end of the chromosomes, hundreds of base pairs are lost each round of replication. Telomeres protect the chromosomes from degradation and damage, and they are necessary for cell proliferation. In mammals, telomeres consist of a six nucleotide tandem repeat, 5′-TTAGGG-3′ (Moyzis et al., 1988). The ribonucleoprotein enzyme telomerase consists of three subunits that are responsible for extending the telomeric repeats (Cohen et al., 2007). Human telomerase RNA (hTERC) and dyskerin are constitutively expressed in cells, but the third subunit, known as human telomerase reverse transcriptase (hTERT), is the limiting factor in telomerase functionality (Shay and Bacchetti, 1997; Li et al., 2003). During each round of mitosis, in the absence of telomerase, approximately 300 base pairs of DNA are lost from the ends of the chromosomes. When telomeres become critically short the cells enter cellular senescence or die via the apoptotic pathway.

Human telomerase reverse transcriptase is under strict transcriptional control in most somatic cells, but this transcriptional control appears to be relaxed in cancer cells, germinal cells, and other self-renewing tissues (Liu et al., 2004). hTERT is the major catalytic subunit for human telomerase, and it specifically facilitates the addition of nucleotides to the 3′ end of a telomere (Nugent and Lundblad, 1998). The reverse transcriptase portion of TERT is highly conserved between species, and mutations to this portion result in loss of function (Lingner et al., 1997). The presence of aspartate residues gives the hTERT subunit an overall negative charge, which can lead to recruitment of metal ions for stabilization. This feature is common in reverse transcriptase enzymes, as the metal ions aid in nucleotide addition (Steitz, 1998). Aberrant expression of hTERT in cancer cells provides a means for escaping cellular senescence and death.

**FIGURE 1:**
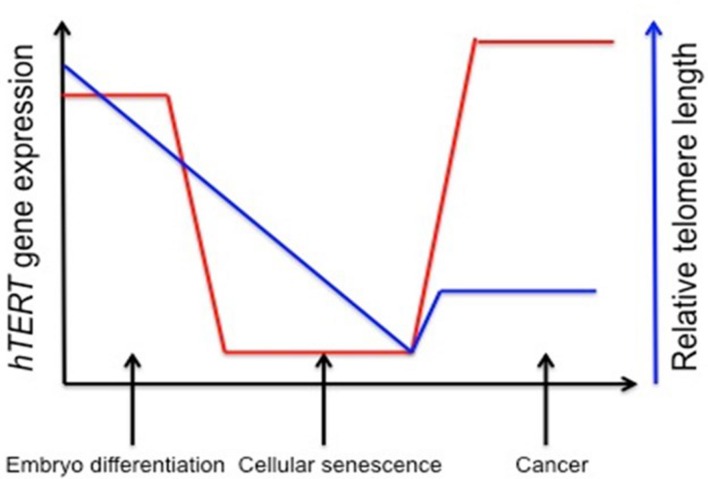
**Stylistic depiction of general trends in human telomerase reverse transcriptase (*hTERT*) gene expression and telomere length during embryogenesis, senescence, and cancer.** *hTERT* gene expression is elevated in embryo development and initial differentiation, but is nearly lost in somatic cells and replicative senescence whereas in tumor cells there is a reactivation of *hTERT*. Telomere length is stable in embryonic stem cells and germline cells, but telomeres begin to shorten during embryonic development until cells reach senescence and crisis. Aberrant reactivation of telomerase activity in cancer allows cells to escape crisis and achieve indefinite proliferation despite telomeres being notably shorter. One explanation for decreased telomere length despite increased hTERT expression is that telomerase cannot keep up with the highly proliferative state of cancer cells (Levy et al., 1992; Tollefsbol and Andrews, 2001).

Telomerase activity is regulated by mechanisms that affect its catalytic activation. Forced expression of TERT is generally enough to induce enzyme activation, and senescence can be bypassed. In many cancers, up-regulating *TERT* mRNA expression and down-regulating tumor suppressor genes such as *Rb* and *p16* can achieve immortality (Wright et al., 1989). Approximately 90% of all human cancers contain an increased level of telomerase (**Figure 1**), and understanding the epigenetic regulation of the gene that encodes for the TERT subunit provides a mechanism for controlling its expression (Kim et al., 1994).

The field of epigenetics provides a modified approach for transcriptional control. There are four major epigenetic mechanisms for gene regulation: DNA methylation, histone acetylation, histone methylation, and non-coding RNA. DNA methylation involves modifying cytosine into 5′-methylcytosine within CpG sites. These CpG sites may cluster as CpG islands, which occur within greater than 50% of gene promoters (Vavouri and Lehner, 2012). Generally DNA methylation is associated with gene repression by preventing activating transcription factors from binding to the DNA. DNA methylation can act as an activator when avoiding the binding of repressors, though (Wan and Bartolomei, 2008). DNA methylation of CpG islands within the promoter is associated with repression, while tissue-specific methylation occurs at CpG ‘shores,’ where there’s a lower CpG density close to the CpG islands. In general, CpG islands are non-methylated in normal cells. Within gene bodies, methylation of expressed genes probably prevents incorrect transcriptional initiation (Barter et al., 2012). Histone acetylation of H3 and H4 is associated with euchromatin, and the acetylation of these histones proximal to a promoter region is associated with gene expression (Schübeler et al., 2004; Vaissiére et al., 2008). Methylation of histones generates a varied response based on the amount of methyl groups added, and to which lysine residue they are added. They can be an indicator of heterochromatin or of active gene transcription. Non-coding RNAs are associated with carcinogenesis through interaction with oncogenes, and by down-regulating tumor suppressors, usually through interaction with the 3′UTR of the mRNA (Gaur et al., 2007; Bartel, 2009). Together these epigenetic gene regulators influence *hTERT* gene expression.

## Organization of the *hTERT* Promoter

The hTERT gene is comprised of 16 exons and 15 introns, spanning ∼35 kb on chromosome 5p15.33 (Bryce et al., 2000). By performing deletion mutagenesis and DNA footprinting it was determined that the promoter region spans from 330 bp upstream of the transcription start site to the second exon (Cong et al., 1999). Promoter activity correlates with telomerase activity, and therefore justifies the assumption that regulation of telomerase is mainly at the transcriptional level.

Takakura et al. (1999) cloned the 5′ promoter region of the *hTERT* gene for the first time in order to map sites of transcription factors. The proximal 260 bp region is identified as the core promoter region, specifically for cancer transcriptional activity. E-boxes, which are binding sites for c-Myc and Mad1, are found at -165 and +44. The consensus sequence of the E-box is 5′-CACGTG-3′. When bound by c-Myc these are key activators for transcription, while Mad1 binds antagonistically to c-Myc at the E-boxes, and serves to suppress *hTERT* gene activity (Tollefsbol and Andrews, 2001). Myc-expressing cells have telomerase activity comparable to that of cancerous cells (Wang et al., 1998). Also present within the core *hTERT* promoter are GC-boxes, which are binding sites for Sp1 transcription factor. Sp1 can interact with *c-Myc* and stimulate telomerase expression through the transcriptional ability of MBD1-containing chromatin-associated factor 1 (MCAF1) (Liu et al., 2007, 2008; Kyo et al., 2008). Further involvement of Sp1 and *hTERT* expression are explored in Daniel et al. (2012). Mutations in any of the five GC-boxes reduce core promoter activity (Kyo et al., 2008). Other key binding sites found in the *hTERT* promoter include AP1, which binds the Jun/Fos dimer as a transcriptional repressor, AP-2, which shows tumor-specific *hTERT* upregulation, and HIF-1, which upregulates *hTERT* expression in hypoxic events. Mutations that generate an ETS binding site play a role in increasing *hTERT* promoter activity (Huang et al., 2015).

**FIGURE 2:**
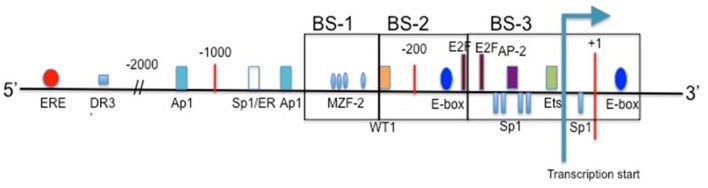
**The promoter region of hTERT.** Included are the binding sites for common transcription factors in the proximal region, and estrogen receptor in the upstream region. Transcription start site is indicated, and translational start occurs at the ATG present at +1 (Cong et al., 1999; Zinn et al., 2007).

Upstream of the core promoter are a high concentration of binding sites for transcription factors as well as hormone response elements. Estrogen and progesterone receptor binding sites are included in this region, which play a large role in telomerase modulation for certain cancers, such as those of the prostate and breast. Other upstream promoter elements may contribute to the regulation of *hTERT*, just in different cells and/or growth conditions (Cong et al., 1999). For a basic schematic of the *hTERT* promoter region see **Figure 2**.

## DNA Methylation and *hTERT* Expression

The epigenetic process of DNA methylation is crucial in gene expression. Methylation occurs genome-wide at CpG sites, usually in non-coding regions, and clusters of CpG sites form islands. These islands tend to be unmethylated and located within gene promoters. There are several DNA methyltransferases (DNMTs) that catalyze the methylation of DNA. These include DNMT3A, DNMT3B, and DNMT1 (Ting et al., 2006). DNMT3A and B are involved in *de novo* methylation while DNMT1 is responsible for the methylation of hemimethylated DNA during the replication process (Irvine et al., 2002). The loss of DNMT3A impairs hematopoietic stem cell (HSC) differentiation, while the loss of DNMT3B results in hypomethylation of certain gene promoters (Challen et al., 2011; Micevic et al., 2016). The hypomethylation of DNMT3B can activate the transcription of microRNAs involved in tumorigenesis.

Canonically DNA methylation is associated with gene silencing. Cancer cells experience variations in DNA methylation status, where the CpG sites often become hypomethylated, and some CpG islands are prone to hypermethylation. This hypermethylation is associated with gene silencing of tumor suppressors such as *p16* and *hMLH1* (a part of DNA mismatch repair) (Merlo et al., 1995; Herman et al., 1998; Li et al., 2013; Feng et al., 2015). *hTERT* is an exception to this rule, though, considering that the majority of the *hTERT* promoter region contains hypermethylated CpG islands in most cancer cells where it is expressed. Methylation status can vary among cell lines. Previous studies determined that CpG methylation correlated inversely with gene expression, but partial methylation of the *hTERT* promoter could exist in telomerase-positive cells (Dessain et al., 2000). Hypermethylation decreases the affinity of transcriptional activators for the *hTERT* promoter region, while hypomethylation allows for binding of transcriptional repressors.

For the *hTERT* promoter, though, this hypermethylated state can prevent transcriptional repressors from binding, such as CTCF. CTCF binds to the first exon of *hTERT* when the CpG island is not methylated (Renaud et al., 2007). This repression of CTCF binding is well reported in the H19/IGF2 cluster (Wan and Bartolomei, 2008). The active *hTERT* promoter is non-methylated at the 11th, 12th, 19th and 27th CpG sites (Choi et al., 2010). These sites of non-methylation are the binding sites for three activators: two SP1 molecules at sites 11, 12, and 19, and one c-Myc protein at site 27 (**Figure 2**). There are three regions within the *hTERT* promoter for methylation analysis. Upstream region from -650 through -400 is considered BS-1, -400 through -150 BS-2, and -150 through +150 is considered BS-3 (with +1 being ATG) (**Figure 2**). Colon cancer cell lines Caco-2 and RKO experience dense methylation in both BS-1 and BS-2, while they only experience partial methylation in BS-3. SW480 colon cancer cells and MCF-7 breast cancer cells experience similar methylation patterns. HCT116 colon cancer cells and H209 lung cancer cells are densely methylated in BS-1 and BS-2, but are completely non-methlyated in BS-3. MDA-MB-231 breast cancer cells experience few non-methylated alleles in BS-3, and are densely methylated in BS-1 and BS-2. MDA-MB-435 breast cancer cells and H82 lung cancer cells are densely methylated in BS-1, slightly methylated in BS-2, and partially methylated in BS-3. Despite varying methylation patterns within these regions, all of the studied cell lines show some degree of non-methylation in BS-3 where the transcription start site is located (Zinn et al., 2007). Despite MDA-MB-231 cells showing increased methylation in BS-3 there must be some present in order for the transcription machinery to bind. *hTERT* promoter methylation can be heterogeneous, and some alleles lack methylation around transcription start site (Renaud et al., 2007).

The degree to which the *hTERT* promoter is methylated plays a role in carcinogenesis. There is a strong association between *hTERT* hypermethylation and gastric cancer, but not between hypermethylation and *hTERT* expression (Gigek et al., 2009). This same result was observed in cervical cancer, as well as ovarian cancer (Widschwendter et al., 2004; Oikonomou et al., 2007). For other cancer types, such as B-cell lymphocytic leukemia, colorectal, and pancreatic cancers the level of *hTERT* methylation impacts telomerase activity (Bechter et al., 2002; Choi et al., 2007; Kumari et al., 2009). Both DNA methylation and histone modification appear to play a role in *hTERT* regulation in hepatocellular carcinoma (Iliopoulos et al., 2009). The multiple methods of *hTERT* regulation and control are proof that other factors play a key role in telomerase activity.

**FIGURE 3:**
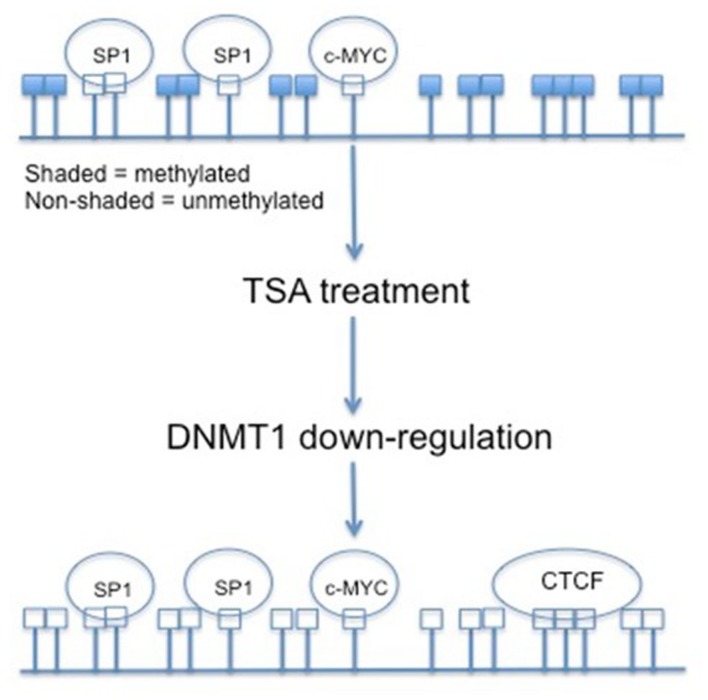
**The effects of TSA on CpG island methylation within the *hTERT* promoter region.** By treating cancer cells with TSA, and in return down-regulating DNMT1, the CpG islands within the *hTERT* promoter are demethylated. This demethylated status allows for the binding of transcriptional repressor CTCF.

Various antitumor agents utilize the fact that the *hTERT* promoter region is hypermethylated in most tumor cells. Trichostatin A (TSA) is a histone deacetylase (HDAC) inhibitor. Transcriptionally active genes in normal human cells treated with TSA will reactivate *hTERT*, but in cancerous cells TSA represses the expression of *hTERT*. This indicates that *hTERT* is under other epigenetic controls. Several CpGs within the *hTERT* promoter become non-methylated upon treatment with TSA. The HDAC inhibitor deacetylates the histones of the DNMT1 gene and decreases its transcription (**Figure 3**) (Choi et al., 2010). The non-methylation associated with TSA treatment results in the opening of CTCF binding sites, and the transcriptional repression of *hTERT* (Ou et al., 2007). Initial methylation status does play a role in TSA effects, though. Cell lines with partially demethylated or non-methylated CpGs within the *hTERT* promoter do not undergo down-regulation associated with TSA treatment (Choi et al., 2010).

5-aza-2′-deoxycytidine (5-azadC) is a common DNA demethylating agent involved in the reexpression of *hTERT* in *hTERT*-negative cells. Demethylation by 5-azadC restores the binding capability of CTCF to the first exon of *hTERT* and E2F-1 to the promoter. Therefore, one of the main roles of *hTERT* methylation is probably to prevent binding of the CTCF and E2F-1 repressors and permit transcription (Kitagawa et al., 2000; Crowe et al., 2001; Guilleret and Benhattar, 2003; Kumakura et al., 2005; Renaud et al., 2007). Hypermethylation of the *hTERT* promoter during senescence is linked to diminished telomerase activity, as well as *hTERT* mRNA expression. Exposing these cells to 5-azadC restores *hTERT* expression (Shin et al., 2003).

Certain proliferative somatic cells experience telomerase activity, such as the colorectal crypts, gastric cells, and endometrial cells. Colorectal cancer cells experience a higher level of methylation within the *hTERT* promoter. Lower methylation within a tumor cell *hTERT* promoter correlated to shorter telomeres and lower telomerase activity (Valls-Bautista et al., 2011). Colorectal tumors with a high degree of *hTERT* promoter methylation revealed high telomerase activity. *hTERT* promoter methylation is required for *hTERT* expression, and thus telomerase activity. For the normal proliferative colorectal cells, though, *hTERT* methylation is not sufficient to sustain telomerase activity. Aberrant methylation of CpG islands within the *hTERT* promoter in addition to a large change in telomerase activity occurs in tumor cells (Kim et al., 2006).

A partially methylated *hTERT* minimal core promoter along with a methylated exon 1 *in vitro* exhibits similar levels of hTERT, as seen in human cancers. Tissue-specific factors play a role in the expression of *hTERT* aside from the methylation status, based on the variation in methylation among cancer cell lines and highly proliferative cells. For example, in adult gliomas, DNA methylation appears to be an alternative mechanism for *TERT* upregulation behind mutations, while pediatric brain tumors experience DNA hypermethylation of the *TERT* promoter (Arita et al., 2013; Castelo-Branco et al., 2013). Consistent with the pediatric study, there was an increase in *hTERT* promoter methylation in adult pituitary adenomas (Köchling et al., 2016). This further demonstrates variation in methylation status among age group and cancer type.

Hydroxymethylation is a transition product for the active loss of methylated DNA, as seen in the comparisons between pluripotent and differentiated cells (Baylin and Jones, 2011). Hydroxymethylation (5′hydroxymethylcytosine, 5 hmC) was first described Kriaucionis and Heintz (2009) and Tahiliani et al. (2009) as the intermediary between methylated and non-methylated DNA. The 10–11 translocation (TET) proteins are responsible for converting 5-methylcytosine to 5-hydroxymethylcytosine, as well as further oxidations to 5-formylcytosine and 5-carboxylcytosine. 5 hmC is linked to transcriptionally active genes and enhancer regions associated with these genes. In this same mechanism, 5 hmC can also be linked to insulator regions, and therefore, transcriptional repression. The TET proteins are responsible for both the activation and repression associated with 5 hmC (Wu and Zhang, 2011). Further mechanisms behind 5 hmC are supported in Dawson and Kouzarides (2012). 5 hmC is highly tissue-specific in normal cells, and the same can be expected for cancerous cells. TET mutations in conjunction with deregulation of epigenetic modifiers can prevent any sort of pattern forming in sites of DNA methylation (Dolnik et al., 2012; Jeschke et al., 2016).

## Histone Modification Effects on *hTERT* Expression

Histones are responsible for chromatin organization with the nucleus of cells. Affecting the charges on the amino acid tails of the histones can change the affinity of the histones for the associated DNA. Histone tail modifications include acetylation, methylation, phosphorylation, and ubiquitination (Jenuwein and Allis, 2001). Most commonly, methylation at lysine 4 on histone 3 (H3K4) and hyperacetylation of histones are associated with active gene transcription, and typically unmethylated DNA. Methylation at lysine 9 and lysine 27 on histone 3 (H3K9 and H3K27) and hypoacetylation are associated with inactive and typically hypermethylated DNA.

Cancer cell lines experience an enrichment of acetyl-H3K9 and dimethyl-H3K4. These are marks for active gene transcription. In contrast, trimethyl-H3K9 and H3K27 are depleted in cancer cells, and are marks for inactive gene transcription. Me-H3K9 (methylated lysine 9 on histone 3) is expressed the lowest in cell lines expressing high levels of *hTERT*. Traditionally repressive Me-H4K20 is observed in similar levels between normal fibroblasts and hTERT-expressing tumor cells, which means that this modification must have an additional role other than gene repression. Tumor cells expressing the highest levels of *hTERT* also express the highest levels of AcH3, AcH4, and Ac-H3K9. Me-H3K4 is also higher in *hTERT*-expressing tumor cells (Liang et al., 2004; Atkinson et al., 2005). These modifications are tightly linked to the promoter sequences and are related to either gene expression or repression.

Human telomerase reverse transcriptase expression can be reactivated by treatment with HDAC and DNMT inhibitors like TSA and 5-azadC, respectively, depending on the cell context. Together these compounds can maintain histone acetylation and DNA demethylation (Cong and Bacchetti, 2000). Telomerase reactivation in telomerase-negative cells can be achieved by chromatin remodeling, such that the promoter region of *hTERT* is more accessible. Myc:Max complexes activate transcription by binding to E-boxes, but these sites are often being competed for by the Mad:Max repressor complex. Mad represses the *hTERT* promoter through the interaction of HDACs (Laherty et al., 1997). This complex can be repressed, though, by chromatin condensation through HDAC inhibitors (Cong and Bacchetti, 2000).

SET and MYND domain-containing protein 3 (SMYD3) is a H3K4-specific dimethyltransferase and trimethyltransferase that plays an important role in oncogenesis, as noted by its upregulation in colorectal carcinoma, hepatocellular carcinoma, and breast cancer (Tsuge et al., 2005; Hammamoto et al., 2006). SYMD3 activates transcription by interacting with its binding motif 5′-CCCTCC-3′ within the promoter region of its target genes, then dimethylating or trimethylating H3K4. The methylated histone increases accessibility of DNA to transcription machinery (Hammamoto et al., 2004). There are five potential SMYD3 binding sites present within the core *hTERT* promoter. A highly trimethylated H3K4 is associated with an actively transcribed *hTERT* gene in telomerase-positive tumor cells (Atkinson et al., 2005). By knocking down SMYD3 with siRNA, there was a significant reduction in *hTERT* mRNA in colorectal carcinoma HCT116 cells, hepatocellular carcinoma Hep3B cells, and Hodgkin’s lymphoma L1236 cells. Two of the SYMD3 binding sites were important for transcription of *hTERT*. Upon knocking down SMYD3, H3K4 trimethylation was abolished in the core promoter, which indicates SMYD3’s responsibility for H3K4 trimethylation specifically within the *hTERT* promoter. Associated with transcriptional repression due to the knockdown of SMYD3 is the inability of both c-Myc and Sp1 to bind the promoter (Guccione et al., 2006; Liu et al., 2007). H3K4 trimethylation is crucial in permitting chromatin accessibility by transcription factors. It is also proposed that E2F-1, a transcriptional repressor of *hTERT*, is involved in the transcriptional activation of SMYD3. The presence of E2F-1 activates SMYD3, which in turn compensates for the inhibitory effect of E2F-1 on the *hTERT* promoter (Tsuge et al., 2005).

As aforementioned, Trichostatin A (TSA) is a HDAC inhibitor involved in the activation of the *hTERT* promoter. Sp sites within the *hTERT* promoter may be important for the HDAC-mediated transcriptional repression within normal human somatic cells. By mutating these sites promoter activity is increased, and there is a decrease in TSA-responsiveness. Endogenous Sp1 and Sp3 are tightly associated with HDAC within the *hTERT* promoter of normal human somatic cells. TSA may convert the Sp1 and Sp3 sites within the promoter from repressor to activator sites through preventing the normally associated HDAC activity (Won et al., 2002). Histone deacetylation is a factor largely responsible for the repression of the *hTERT* within normal human somatic cells (Xu et al., 2000). Many HDAC inhibtors and demethylating agents, including TSA are responsible for down-regulating *hTERT* in leukemia cells on an epigenetic level (Sui et al., 2013).

Suberoylanilide hydroxamic acid (SAHA), clinically known as vorinostat, is also a HDAC inhibitor (Bouchain and Delorme, 2003). It has various anticancer affects, and is clinically approved for the treatment of cutaneous T-cell lymphoma (CTCL) by inducing apoptosis (Zhang et al., 2005). SAHA can also induce cell cycle arrest and inhibition of differentiation in cancer cells (Munster et al., 2001; Arnold et al., 2007). SAHA has an indirect effect on the methylation status of the *hTERT* promoter via down-regulation of DNMT1 and DNMT3b, and, therefore, induces demethylation of the CpGs in non-small cell lung cancer cells (Li et al., 2011).

Sirtuin 1 (SIRT1) is an NAD-dependent deacetylase involved in telomeric maintenance, and may act as a tumor promoter or suppressor depending on the type of cancer in which it is implemented (Fang and Nicholl, 2011). SIRT1 is elevated in prostate tumors and hepatocellular carcinomas (Huffman et al., 2007; Chen et al., 2011, 2012; Choi et al., 2011). SIRT1 does not regulate *hTERT* through the proximal promoter, it did not regulate *hTERT* through the 3′UTR, and it may not affect *hTERT* expression through CpG island methylation. Based on this information, SIRT1 interacts with transcription factors associated with the *hTERT* promoter. Depletion of SIRT1 is associated with H3K9 acetylation and reduction of H3K9 trimethylation of the *hTERT* promoter (Zhang et al., 2014). SIRT1 physically interacts with the C-terminus of c-Myc and deacetylates it. This deacetylation causes c-Myc to associate with Max, thus facilitating its activity on the *hTERT* promoter (Mao et al., 2011).

## Non-Coding RNA and *hTERT*

**FIGURE 4:**
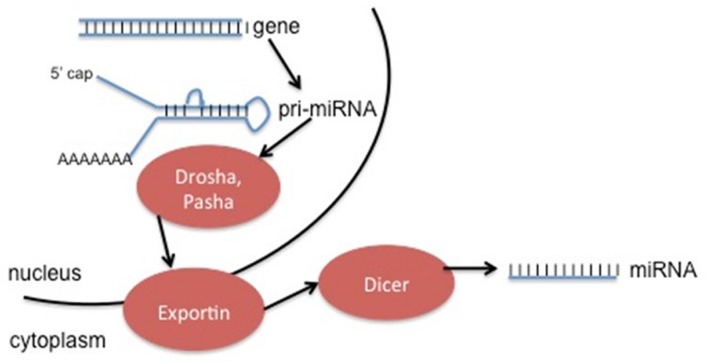
**Generation of ncRNA can occur from specific genes (as seen above), or by modification of excised introns.** The method depicted above involves initial transcription of the gene into pri-miRNA, followed by cleavage into pre-miRNA by the enzymes Drosha and Pasha. Exportins move the pre-miRNA into the cytoplasm, where the enzyme Dicer removes the hairpin loop in order to generate the final miRNA product. This product can act alone or be incorporated into an enzyme complex.

Non-protein coding RNA molecules are a rapidly growing class of RNA molecules that regulate the activity of specific mRNA. MicroRNAs (miRNAs) are approximately 18–25 nucleotides in length, and expression is strongly associated with disease progression, including cancers (Kala et al., 2013). These miRNA molecules can be used as diagnostic and prognostic biomarkers in cancer type and progression (Grady and Tewari, 2010). miRNA is generated in a complicated process (**Figure 4**), and can be found not only in the introns or exons of genes, but also in intergenic sequences (Bartel, 2004). Deregulation of these non-coding RNAs has the ability to contribute to cancer formation by acting with oncogenes and/or down-regulating tumor suppressors (Gaur et al., 2007). Usually miRNAs play an important role in post-transcriptional regulation of target genes by binding recognition sites in the 3′ untranslated regions (3′UTRs) of transcripts, specific base pair sequences within the 5′UTRs, and ORFs (Bartel, 2009).

miRNAs have the ability to regulate the expression of epigenetic-modifying enzymes involved in carcinogenesis, as well as genes that play a role in chemoresistance (Guil and Esteller, 2009). Specific non-coding RNA interaction with *hTERT* has been found in multiple types of cancers. The miRNA profile is different for each cell type, but *miR-491-5p* has been shown to be involved in the initiation and progression of multiple tumor types, including cervical cancer. It is down-regulated in cervical cancer, and enforced expression of *miR-491-5p* significantly inhibited proliferation through targeting *hTERT* (Zhao et al., 2015). *miR-1182* modulates *hTERT* protein levels by binding the open reading frame of *hTERT* mRNA between 2695 and 2719 within different types of gastric cancer cells. Upon examining tissues of patients with gastric cancer, there was an inverse correlation between *miR-1182* and *hTERT*, which shows *miR-1182* as a potential treatment of gastric cancer (Zhang et al., 2015). *miR-1207-5p* and *miR-1266* also target *hTERT* within gastric cancers, and are significantly repressed in gastric cancer tissue samples (Chen et al., 2014). Overexpression of *miR-138* induced a reduction in hTERT protein expression by interation with the 3′UTR in anaplastic thyroid carcinoma (ATC) (Mitomo et al., 2008). Other microRNAs that directly regulate TERT include *let-7g*, *miR-133a*, -*342*, and -*541* (Hrdličková et al., 2014). Directly regulating *hTERT* mRNA with miRNA will become more feasible as more of these non-coding RNAs are discovered. A summary of *hTERT* regulation by miRNA can be seen in **Table 1**.

**Table 1:** Regulation of *hTERT* via miRNA.

miRNA	Tissue type	Mode of action	Reference
miR-491-5p	Cervical cancer	Unknown, inhibits *hTERT*	Zhao et al., 2015
miR-1182	Gastric cancer	Binds the ORF of *hTERT* mRNA, preventing translation	Zhang et al., 2015
miR-1207-5p	Gastric cancer	Represses *hTERT* in normal tissues	Chen et al., 2014
miR-1266	Gastric cancer	Represses *hTERT* in normal tissues	Chen et al., 2014
miR-138	Anaplastic thyroid carcinoma (ATC)	Interaction with 3′UTR of *hTERT* to reduce protein expression	Mitomo et al., 2008
let-7g	Pulmonary fibrosis	Interaction with 3′UTR of *hTERT* to reduce expression	Singh et al., 2010
miR-133a	Jurkat cells	Interaction with 3′UTR of *hTERT* to reduce expression	Hrdličková et al., 2014
miR-342	Jurkat cells	Interaction with the 3′UTR of *hTERT* to reduce expression	Hrdličková et al., 2014
miR-541	Jurkat cells	Interaction with the 3′UTR of *hTERT* to reduce expression	Hrdličková et al., 2014


Non-coding RNAs can also target transcription factors involved in the control of *hTERT*. *miR-21* targets *E2F*, *miR-26*, -*107*, and -*210* are induced in response to low oxygen via HIF-dependent mechanisms, and decrease proapoptotic signaling within the hypoxic environment (Chan et al., 2005; Kulshreshtha et al., 2007). In pancreatic cancer, *miR-494* was down-regulated and correlated with poor prognosis. c-Myc and SIRT1 expression levels were inversely correlated with *miR-494* expression in pancreatic cancer tissues due to direct interaction with the 3′UTR with the mRNA transcripts of both c-Myc and SIRT1, and restoring *miR-494* sensitized the cells to chemotherapy (Liu Y. et al., 2015). c-Myc is a direct target of *miR-1294* in esophageal squamous cell carcinoma. Down-regulating *miR-1294* directly resulted in a poor prognosis by elevating c-Myc expression (Liu K. et al., 2015). These are just a few examples of non-coding RNA epigenetic control of hTERT indirectly. Each day more microRNAs are being discovered and characterized for their role in gene regulation.

Long non-coding RNAs (lncRNAs) are also emerging in association to gene regulation and cancer. Their regulatory function is much more extensive than that of miRNAs, which involves competing endogenous RNAs (ceRNAs) (Yamaguchi and Abe, 2012). lncRNA, ceRNA, and mRNA transcripts can affect each other by competing for the miRNA response element (MRE) (Cesana et al., 2011; Salmena et al., 2011; Shi et al., 2013). The lncRNAs compete for miRNAs, and inhibit their binding to MREs, therefore, protecting target RNAs. lncRNAs and *hTERT*-encoding genes are syntropic transcripts and may influence adjacent gene expression. For example, lncRNA BC032469 is overexpressed in gastric cancer tissue, and its expression is correlated with tumor size and differentiation, as well as hTERT protein abundance (Lü et al., 2015).

## Dietary Compounds and Their Effect on the *hTERT* Promoter

There has been much interest recently in the consumption of dietary compounds in order to make reversible changes to the DNA. This concept has been coined the “epigenetics diet”, and utilizes bioactive dietary components to cause changes to the epigenome (Hardy and Tollefsbol, 2011). Some examples of these foods include green tea (tea polyphenols), soybeans (genistein), grapes (resveratrol), cruciferous vegetables (sulforaphane), and turmeric (curcumin). These components have the ability to alter the status of DNA methylation and histone modification. Epigenetic modifications by bioactive compounds can induce tumor suppressor genes or inhibit tumor-promoting genes (Meeran et al., 2010a). These phytochemicals can also influence the expression of non-coding RNAs. They affect the expression of different miRNAs depending on the cancer type (Krakowsky and Tollefsbol, 2015). An important benefit for bioactive compounds is the ability to induce gene regulation and apoptosis selectively in cancer cells, but not in normal cells.

### Tea Polyphenols

The most prevalent chemical compound in green tea is the family of catechins, the most abundant being (-)-epigallocatechin-3-gallate (EGCG) (Graham, 1992; Lin and Liang, 2000). In general, the epigenetic anticancer effects of EGCG include the inhibition of DNMT1, leading to demethylation and reactivation of methylation-silenced genes, although there are notable exceptions (Kim et al., 2010; Chen et al., 2011; Shanmugam et al., 2011). EGCG implements its inhibitory effects by blocking cytosine from entering DNMT1’s active site (Fang et al., 2003). Because a hypermethylated promoter activates *hTERT*, EGCG has the ability to inhibit telomerase activity by demethylating the *hTERT* promoter. The demethylation allowed transcriptional repressors such as E2F-1 to bind (Berletch et al., 2008). EGCG also has strong histone acetylase (HAT) inhibitory activity, and can modify gene expression by histone modifications (Choi et al., 2009). EGCG can remodel the chromatin associated with the *hTERT* promoter by decreasing acetyl-H3, acetyl-H3K9, and acetyl-H4. Hypomethylation and deacetylation recruits transcriptional repressors such as E2F-1 and Mad1 (Meeran et al., 2011).

### Genistein

Genistein is an isoflavone/phytoestrogen found in soybeans, fava beans, kudzu, lupin, and psoralea (Valls et al., 2009). Genistein is responsible for reactivating tumor suppressor genes such as *p21*, *p16*, and *BTG3* by DNA demethlyation and histone modifications (Kikuno et al., 2008; Majid et al., 2008). It also inhibits the expression of *hTERT* in breast cancer cells by inihibiting DNMT1, DNMT3a, and DNMT3b, and trimethylating H3K9. Dimethyl-H3K4, an active transcription marker, is depleted in response to genistein, and hypomethylation of the E2F-1 recognition site causes increased binding to the *hTERT* promoter. Binding of c-Myc decreases in response to genistein. By combining genistein with 5-azadC there was a higher *hTERT* inhibition than that of each treatment alone (Li et al., 2009). Because genistein is a phytoestrogen, it can be found more highly concentrated in breast tissues with a greater distribution of estrogen receptors (Cassidy and Faughnan, 2000). Depending on the tissue type and tumor stage, though, genistein can also have very negative effects (Balabanič et al., 2011; Schug et al., 2011; Guerrero-Bosagna and Skinner, 2014).

### Resveratrol

Resveratrol is a polyphenol derived from grapes, berries, peanuts, and other plant sources, but is most commonly consumed in the form of red wine. Its anticancer properties include the ability to inhibit proliferation of human tumor cells through a variety of epigenetic mechanisms. Resveratrol has weaker anti-DNMT activity compared to some of the other bioactive compounds, and is associated with activation of type III HDAC inhibitors, such as SIRT1 and p300 (Howitz et al., 2003; Kaeberlein et al., 2005). The activation of SIRT1 by resveratrol decreases expression of the anti-apoptotic protein Survivin by deacetylating H3K9 within its promoter (Stünkel et al., 2007; Wang et al., 2008). Treatment of cells that had undergone oncogenic events with resveratrol *in vitro* actually shows an increase in telomerase activity (Pearce et al., 2008; Wang et al., 2011). Studies from our laboratory have shown that resveratrol in combination with pterostilbene suppresses *TERT* activity via suppression of SIRT1, γ-H2AX, and DNMTs within breast cancer cells (Kala et al., 2015).

### Sulforaphane (SFN)

Sulforaphane (SFN) is an isothiocyanate that is abundant in cruciferous vegetables including, but not limited to, broccoli, cauliflower, cabbage, and kale. SFN is a DNMT inhibitor, and treatment exhibits both a dose- and time-dependent inhibition of *hTERT* in MCF-7 and MDA-MB-231 breast cancer cells. SFN induced site-specific demethylation of CpG islands within the first exon of *hTERT*, which facilitated CTCF binding, and thus repression of transcription. Repression of *hTERT* was followed by apoptosis in breast cancer cells (Meeran et al., 2010b). SFN also has HDAC inhibitory activity in HCT116 colorectal cancer cells, prostate cancer BPH-1, LNCaP, and PC-3 cells (Myzak et al., 2004, 2006; Ho et al., 2009).

### Curcumin

Curcumin is a yellow pigment found in turmeric, and it has been extensively studied for its anti-oxidant, anti-inflammatory, and anti-cancer properties (Sharma et al., 2005). It is a known DNMT inhibitor and HDAC inhibitor, but its solubility and bioavailability are proven obstacles in being a viable therapeutic (Aggarwal et al., 2003; Shishodia et al., 2007; Fu and Kurzrock, 2010). Curcumin inhibits telomerase activity in T47D human breast cancer cells, and it down-regulates the expression of *hTERT* mRNA (Khaw et al., 2013; Nasiri et al., 2013). Curcumin exhibits its inhibitory effect by binding to the catalytic thiolate of C1226 of DNMT1, preventing DNA methylation, and resulting in global hypomethylation (Liu et al., 2005). Depending on the type of cancer cell, curcumin has a strong antagonistic effect on HDACs and HATs. CBP and p300 are two HATs targeted by curcumin, which suppresses non-homologous end joining (NHEJ) and homologous recombination (HR) by down-regulation of *BRCA1* (Marcu et al., 2006; Ogiwara et al., 2013). Hypoacetylation of genes is associated with gene silencing.

There are many more bioactive compounds that have been identified for their abilities to cause changes to the epigenome through inhibition of methylation or histone modification. These include more isothiocyanates, selenium, garlic, and folic acid. Dietary factors in which bioactive compounds are found include coffee, cashews, milk, parsley, rosemary, thistle, and tomatoes (Hardy and Tollefsbol, 2011). Combinations of dietary components should also be considered due to their ability to cause synergistic, additive, or antagonistic effects. Together they have the potential to target many of the cellular processes involved in control of the *hTERT* promoter.

## Conclusion

Human telomerase reverse transcriptase is regulated by several modes of epigenetic modifications. These epigenetic changes are proving to be fruitful ways of selectively targeting *hTERT* in terms of prevention or potential cancer treatment. Although telomeres are shorter in cancer cells than normal somatic cells, the enzyme responsible for telomere maintenance, telomerase, is upregulated in approximately 90% of all human cancers. Expression of the telomerase reverse transcriptase subunit of the holoenzyme telomerase is responsible for regulating enzyme activity. The down-regulation of DNMTs reduces the hypermethylated state of the *hTERT* promoter by allowing repressor binding. Chromatin remodeling changes the state of histones present within the *hTERT* promoter by influencing the binding of transcription factors. miRNAs can change the expression of *hTERT* in a post-transcriptional manner by binding to the 3′UTR of its mRNA, or by affecting the presence of transcription factors responsible for the transcription or repression of *hTERT*. Dietary compounds can also influence *hTERT* by increasing or decreasing the activities of DNMTs and histone-modifying enzymes.

Expression of *hTERT* does not appear to be controlled simply by one mechanism. Modes of regulation are cell-type and age specific. Epigenetic modifiers are epigenetically modified, so control of *hTERT* can function indirectly. There is no ‘golden bullet’ responsible for epigenetic regulation of *hTERT*. This is why combinations of epigenetics-altering drugs are used to manipulate telomerase expression. Controlling *hTERT* epigenetically through various mechanisms appears to be a more consistent approach to preventing or treating cancers that have aberrantly expressed telomerase.

## Author Contributions

KL and TT conceived of the review article and participated in all drafts of the manuscript. KL wrote the first draft of the manuscript with guidance from TT. TT performed final editing and approval of the manuscript. All authors read and approved the final draft.

## Conflict of Interest Statement

The authors declare that the research was conducted in the absence of any commercial or financial relationships that could be construed as a potential conflict of interest.
